# Prophylactic Treatment of Pediatric Migraine: Is There Anything New in the Last Decade?

**DOI:** 10.3389/fneur.2019.00771

**Published:** 2019-07-16

**Authors:** Laura Papetti, Fabiana Ursitti, Romina Moavero, Michela Ada Noris Ferilli, Giorgia Sforza, Samuela Tarantino, Federico Vigevano, Massimiliano Valeriani

**Affiliations:** ^1^Department of Neuroscience, Headache Center, Bambino Gesù Children Hospital, Rome, Italy; ^2^Child Neurology Unit, Systems Medicine Department, Tor Vergata University Hospital of Rome, Rome, Italy; ^3^Center for Sensory-Motor Interaction, Aalborg University, Aalborg, Denmark

**Keywords:** migraine, pediatric migraine, prophylactic drugs, therapy, treatment, guidelines, preventive

## Abstract

Migraine is a frequent and very disabling disease, especially at pediatric age. Despite this, there are few controlled data on the prophylactic treatment of primary headaches in this category of age. Given that the recently introduced calcitonin gene-related peptide (CGRP) inhibitors (CGRP-r) are still limited to adulthood, there is no drug with exclusive indication for migraine treatment in pediatric age. This raises several limitations in terms of adherence and effectiveness of the therapy. Moreover, the scenario is complicated by placebo response, which is larger in children and adolescents than in adults and often leads to an improvement in the attack frequency even in absence of any active pharmacological treatment. Our aim was to investigate the real evidence concerning the prophylactic therapy of pediatric migraine by reviewing the clinical studies published between 2010 and 2019.

## Introduction

According to epidemiological studies, the prevalence of headache in children varies from 5.9 to 82% (1). Migraine, the most common type of primary headache in children, is highly disabling even in childhood and adolescence. The average prevalence of pediatric migraine varies according to age, going from 3% in younger children to ~20% in adolescents (2). A noticeable social problem is represented by chronic migraine (more than 15 days with headache a month) that afflicts from 0.6 to 1.8% of children and adolescents (3).

The main reference for the diagnosis of primary headaches are the criteria of the International Headache Society (IHS) (4). These criteria have shown limitations when applied in the pediatric age (1, 5), although the last version (ICHD 3) considers some peculiarities of migraine in pediatric age, such as the shorted duration of pain and the unilateral/bilateral location of pain (1, 5).

Regarding therapies of pediatric migraine, there is a significant lack of clinical studies on acute and prophylactic therapy. This is partly due to differences between countries, where therapeutic approaches are based on cultural and political factors. Few clinical trials are available in pediatric patients and they often show conflicting findings. The paucity of data on the effectiveness of treatments in young migraineurs is also due to the power of placebo effect, in terms of reduction of both frequency and intensity of migraine attacks (6). Though representing a precious resource, the placebo effect can paradoxically represent an obstacle in controlled trials comparing the efficacy of pharmacological and non-pharmacological treatments with placebo.

Migraine prophylaxis aims at reducing the impact of migraine by improving the frequency and intensity of attacks. In children and adolescents, it should be considered when the frequency of attacks is higher than 4 attacks per month or the response to the symptomatic treatment is not satisfactory. In a previous retrospective review, Papetti et al. (7) emphasized the lack of definitive data on the possible drugs to be used.

Here, our aim is to investigate the actual evidence concerning prophylactic therapy of pediatric migraine by reviewing clinical studies published between 2010 and 2019.

## Methods

### Literature Search Strategy

We considered studies published from January 2010 to January 2019. Medline and Cochrane library were used for the research. Search words were: “migraine and treatment or therapy,” “migraine and prophylaxis,” and “migraine and guidelines.” The filters included clinical trials (CT), randomized control trials (RCTs), open label studies (OL), retrospective studies (RS), meta-analysis, multicenter studies, reviews and articles that were either published in the last 10 years. Our search was focused on the age group ranging from 0 to 18 years, although any article that included adult population but contained patients under the age of 18 years was also considered. Two authors (F.U. and L.P.) independently checked the studies identified by the literature search. All potentially relevant studies were reviewed by the two authors.

### Search Results

**Figure 1:**
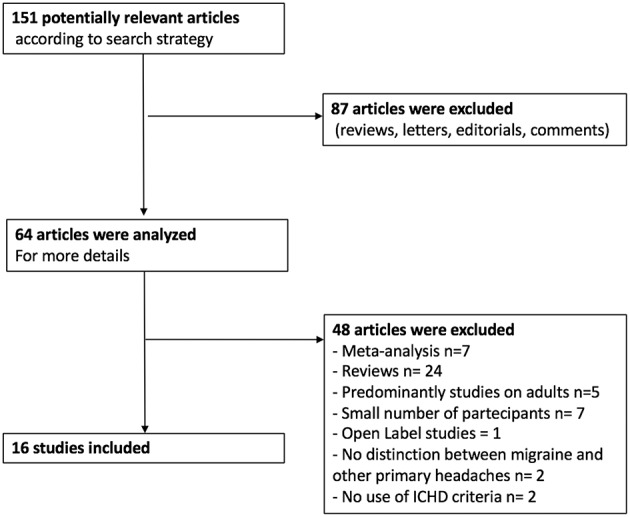
Flow diagram of the study methodology.

Using the above described strategy, 64 articles concerning preventive treatment of migraine in children were included in our study. Among them, there were 40 systematic reviews or meta-analysis of the literature concerning the prophylactic treatment of pediatric migraine, 21 clinical trials (CTs), and 3 retrospective studies (RSs). As for the CTs, 15 were randomized control trials (RCTs) and one was an open label study (OL) (Figure 1). All the included studies were published from 2010 to the present. Results of current evidence are resumed in Table 1.

**Table 1:** List of commonly used drugs for preventive treatment of pediatric migraine.

**Drug pharmacological class**	**Evidence level**	**Dosage**	**Side effects**	**When to be preferred?**
**CALCIUM CHANNEL BLOCKERS**				
Flunarizine	A	5–10 mg/day	Sedation, dizziness, constipation, increased appetite, weight gain Drowsiness, asthenia, weight gain, depression and extrapyramidal symptoms	Associated anxiety and insomnia not overweight patients
**NON-SELECTIVE BETA ADRENOCEPTOR ANTAGONIST**				
Propranolol	C	3 mg/kg/day	Fatigue, reduction of mood, nightmares. Less frequent adverse events: bradycardia, orthostatic hypotension, impotence, hallucinations, weight gain	History of hypertension No history of asthma or allergy No history of bradyarrhythmia
**TRICYCLIC ANTIDEPRESSANT**				
Amitriptyline	B	1 mg/Kg/day	Sedation, dizziness, constipation, increased appetite, weight gain	Not obese patients history of depression or insomnia chronic migraine
**ANTIEPILEPTIC DRUGS**				
Sodium Valproate	B	30 mg/kg/day	Somnolence, nausea/vomiting, thrombocytopenia, tremor, alopecia, increased appetite, emotional lability	History of psychosis Male patients
Topiramate	A	2–3 mg/Kg/day	Paresthesia, somnolence, dizziness, anorexia, metabolic acidosis, cognitive/memory dysfunction	Overweight No history of cognitive impairment
**SEROTONIN MODULATORS**				
Pizotifen	C	1.5 mg/day	Increased appetite, weight gain, drowsiness, sleepiness, dizziness, dry mouth, tiredness, constipation	No obese patients history of depression or insomnia
Cyproheptadine	C	0.2–0.4 mg/kg/day	Drowsiness, fatigue, increased appetite, weight gain, dizziness	No history of asthma
**NUTRACEUTICS**				
Hydroxytryptophan	C	100 mg Kg/day	Nausea, bloating Flatulence, loose stools or diarrhea	Mild intensity of the attack Low frequency Refusal of pharmacological drugs Very young children (<6 year)
Magnesium	C	400–600 mg/day	Nausea, abdominal pain	
Butterbur(petasites hybridus)	C	100–150 mg	Burping or belching Itchy eyes, diarrhea, difficulty breathing, drowsiness, liver toxicity	
Riboflavin	C	400 mg/day	Diarrhea, increased urine	
Coenzyme Q10	C	150–300 mg/day	Nausea and/or vomiting upset stomach, diarrhea heartburn, loss of appetite, abdominal pain or discomfort	
Tenacetum parthenium –Feverfew (MIG99)	C	6.25 mg 18.75 mg TID/day	Abdominal pain, mouth ulcers, bloating, diarrhea, nausea	

*RS, Retrospective Study; RMS, Retrospective Multicenter Study; RCT, Randomized Controlled Trial; TPM, topiramate; PZT, pizotifen; VPA, valproic acid; AMI, amitriptyline; PGB, pregabalin; PPL, propranolol; FNZ, flunarizine; CNZ, cinnarizine*.

## Pharmacological Treatment

### Calcium Channel Blockers

Flunarizine is a calcium channel blocker with properties on the cerebrovascular circulation. How flunarizine acts in preventing migraine is not yet established but it probably has both vascular and neuronal effects (8).

In an RS (2012), Basheer Peer et al. demonstrated that flunarizine (2.5–10 mg/day) shows good efficacy in children and adolescents (median age 13 years), leading to at least a 50% reduction in attack frequency in 57% of patients (41/72). Interestingly, the response rate was particularly high in patients with hemiplegic migraine (85%). The study also showed that flunarizine was well-tolerated with a reasonable safety profile. Side effects were observed in 21% of children and adolescents and included depression, weight gain and sedation (9). In a retrospective study of 475 patients, Kim et al. (10) showed that the efficacy and tolerability of flunarizine 5 mg/day were comparable to those of topiramate. The responder rate (50% reduction in headache days/month) was 80% (89/111 patients) for flunarizine (5 or 10 mg /day) and 81% (122/150 patients) for topiramate (from 25 to 100 mg/day). The frequency of adverse effects was higher in topiramate (10%) than flunarizine (6%) (10). In 2014, Topcu et al. used the PedMIDAS (the score of disability assessment in pediatric migraine) to evaluate the efficacy of different prophylactic therapies in 53 patients, recruited from a series of 88 patients suffering from migraines with an age ranging from 6 to 17 years. They found that topiramate (1–2 mg/Kg/day), propranolol (20–40 mg/day), and flunarizine (5–10 mg/day) significantly decreased PedMIDAS score. The number of days with analgesic treatment significantly decreased in the patients treated with topiramate and propranolol (*p* < 0.05), while it remained unchanged in the flunarizine (*p* > 0.05) (11). More recently, Toldo et al. (12) conducted a retrospective multicenter study among 706 patients with primary headaches. Preventive drugs were used in 19% of migraineurs and in 3% of patients with tension-type headache (12). In patients with migraine, the most used drug was flunarizine (18%), followed by antiepileptic drugs (7%) and pizotifen (6%). Flunarizine and pizotifen were the most effective drugs (72 and 82%, respectively) (12).

Flunarizine is licensed in Italy for patients over 18 years (7) and widely prescribed in Europe, while it is not licensed in the UK or the USA given the lack of published data in the development age. Placebo-controlled clinical trials in pediatric age are needed to confirm its effectiveness in pediatric migraine (13).

### Beta-Blockers

Propranolol is a non-selective beta (b) adrenoceptor antagonist that blocks the b1,2 receptors. Propranolol started to be used in the prophylaxis of migraine for more than 50 years (14). Propranolol showed efficacy and high profile of tolerability in several clinical trials on adult migraine (4). On the contrary, there are only a few studies supporting the efficacy of propranolol in pediatric age (15–17).

In 2010, Bidabadi et al. compared the efficacy and safety of propranolol (started at a dosage of 3 mg/kg/day) and valproate (30 mg/Kg/day) for migraine prophylaxis in childhood. In this study, 60 patients were enrolled (30 in the group A that received propranolol 3 mg/kg/day and 30 in the group B treated by sodium valproate 30 mg/kg/day). The mean age of the patients was 9.85 ± 2.63 years. Headache frequency was significantly reduced by more than 50% in 83% of patients treated with propranolol and in 63% of patients treated with sodium valproate without significant differences between the drugs. Furthermore, no significant difference in side effects between the two groups was found (18). Eidlitz-Markus et al. (19) compared the efficacy of a low dose of propranolol (the initial dose was 0.47 ± 0.17 mg/kg/day) with a low dose of amitriptyline (mean initial dose, 0.26 ± 0.1 mg/kg/day) in children and adolescent suffering from severe migraine. Although the study was not blinded and placebo controlled, it included a large number of patients (118 with a mean age of 12.54 ± 3.14 years). Both propranolol and amitriptyline, when combined with non-pharmacologic treatments, showed efficacy in reducing the frequency of migraine attacks in children (reduction of attack frequency >50% per month in 80% of patients). Propranolol group showed less frequent side effects (19). In 2012, Fallah et al. compared efficacy and safety of propranolol (1 mg/kg/day) and topiramate (3 mg/kg/day) in a parallel single-blinded randomized clinical trial. Authors enrolled 100 patients that were divided in two groups (50 patients treated with propranolol and 50 patients treated with topiramate). After 3 months of treatment, 62% of patients treated with propranolol and 82% of patients treated with topiramate showed more than a 50% reduction in monthly headache frequency (*p* < 0.05). No serious adverse events were seen in both groups and, in particular, the main side effects after treatment with propranolol were mild hypotension and drowsiness (20). In a RCT (2013), Bakhshandeh Bali et al. compared effectiveness, safety and tolerability of propranolol (10 to 20 mg/day divided in two doses; group b) and pregabalin (50 to 75 mg/day; group a). After 4 and 8 weeks of pregabalin administration, headache frequency was reduced by 81.8 and 85.45%, respectively. Using the same treatment intervals, propranolol reduced monthly headache frequency by 64.54 and 68.25%, respectively. The difference between drugs was statistically significant (*p* = 0.04) (21).

Recent data showed that beta-blockers are rarely used in Italy, probably because their tolerability profile is not excellent and they are licensed over 18 years (4).

### Tricyclic Antidepressant

Amitriptyline is one of the most used drugs for preventive treatment of pediatric migraine (22). It is also recommended in cases of tension-type headache associated with anxiety, insomnia and depression (22). Efficacy of amitriptyline prophylaxis is achieved with much lower doses than those required for anti-depressive therapy (10–20 mg/day up to 25–75 mg/day) (7). It is advisable to use increasing doses before reaching the maintenance dose in order to reduce the side effects and improve tolerability. Contraindications are cardiac, hepatic, renal, prostatic and thyroid diseases; glaucoma, hypotension, epilepsy, use of anti-MAO. Amitriptyline also should be used with caution for its anticholinergic effects. The most frequent adverse events are dry mouth, constipation, sedation, and increase in appetite, increased weight, occasionally orthostatic hypotension and cardiotoxicity (22).

As reported above, it was shown that low-dose propranolol and low-dose amitriptyline, if combined with non-pharmacological measures, were both effective in reducing migraine attacks frequency (19). Between July 2012 and November 2014, Hershey et al. conducted a double-blinded, placebo-controlled study with the aim to determine the most effective prophylactic treatment in children and adolescents (CHAMP study). Authors compared the efficacy of amitriptyline, topiramate, and placebo in 361 subjects (from 8 to 17 years of age). In a period of 6 months, 52% of patients receiving amitriptyline (dose 1 mg/kg per day), 55% of patients receiving topiramate (dose 2 mg/kg per day), and 61% of patients receiving placebo had a reduction in headache days of at least 50%, without any significant difference between groups. Furthermore, the patients treated with amitriptyline or topiramate presented higher rates of adverse events compared to placebo control group (23). In conclusion, considering the negative outcome of this study in terms of efficacy and the increased risk of undesirable effects from amitriptyline or topiramate in this sensitive category of patients, the benefit / risk ratio of these drugs is considered unfavorable. In an Iranian parallel, single-blinded randomized clinical trial, the efficacy of amitriptyline (1 mg/kg/day) was compared to melatonin (0.3 mg/kg/day) in a population of migraineurs ranging from 5 to 15 years. A reduction of more than 50% in monthly headache frequency was seen in 82.5 and 62%.5 of patients treated with amitriptyline and melatonin, respectively. Amitriptyline was significantly more effective (*P* = 0.04) (24). Amitriptyline showed a good efficacy for treatment of chronic headaches in association with cognitive behavioral therapy (25–28).

### Antiepileptic Drugs

Sodium Valproate (500–1,500 mg/day) and topiramate (50–100 mg/day) were evaluated for prophylactic therapy of pediatric migraine in some controlled studies (7).

In the last 8 years, one RCT compared the efficacy of valproate and propranolol for the preventive treatment of migraine in the pediatric age. Sixty children (aged 5–15 years) with migraine without aura were included. Patients received propranolol (3 mg/kg/day) or sodium valproate (30 mg/kg/day) for at least 6 months. The main endpoint (reduction of more than 50% in monthly headache frequency) was observed in 83% of the propranolol group and in 63% of sodium valproate group without statistical significance. The global reduction of baseline headache frequency was better in the group of propranolol (*p* < 0.05) (18).

Topiramate is a first-line strategy for the treatment of migraine in adults. In 2014, the U.S. Food and Drug Administration (FDA) approved topiramate for migraine treatment in the pediatric patients aged 12 to 17 years (29). In adults, topiramate proved efficacious in the preventive treatment of migraine with and without aura in episodic and chronic form, and excessive use of symptomatic drugs (24, 30). In a parallel single-blinded randomized clinical pediatric trial, the efficacy and safety of topiramate (3 mg/Kg/day) and propranolol (1 mg/Kg/day) were compared, and the results showed that topiramate was more effective in reducing the monthly frequency, severity, duration and disability of the headache. Topiramate was superior to propranolol in reducing the frequency of the attacks by at least 50% (respectively 82 vs. 62% of patients) (31). In another study by the same authors, recruiting a population of 100 pediatric patients (mean age of 10.46 ± 2.11 years) treated with topiramate (3 mg/kg/day), the frequency and duration of headache attacks reduced from 15.34 ± 7.28 to 6.07 ± 3.16 attacks and from 2.28 ± 1.55 to 0.94 ± 0.35 h, respectively. The pediatric migraine disability assessment score was reduced from 32.4 ± 9.3 to 15.5 ± 6. Side-effects were seen in 21% of the patients, including hyperthermia, anorexia and weight loss, and drowsiness (32). Authors concluded that topiramate could be considered a safe and effective drug for migraine therapy in pediatric patients (32). As reported above, Kim et al. showed that the response rate, retention rate and the rate of side effects were not significantly different between flunarizine and topiramate (10). In a randomized, double-blind clinical study of 44 migraineurs (aged 4–15 years), Ashrafi et al. compared the efficacy and safety of cinnarizine and topiramate in the prevention of pediatric migraine. The primary endpoint was the monthly frequency of migraine. Measures of secondary efficacy were the intensity of monthly migraine and a response rate higher than 50%. During the double-blind phase of the study (week 8), both patients treated with cinnarizine and topiramate showed a statistically significant 50% responder rate (cinnarizine: 55%, *p* = 0.004; topiramate: 50%, *p* = 0.001). Also monthly migraine intensity reduced in both groups (*p* < 0.001) (33). After 12 weeks of treatment, a significant reduction of monthly migraine frequency was observed for both cinnarizine and topiramate (*p* < 0.05) with no significant differences between groups (33).

The CHAMP study failed in showing any superiority of treatment with amitriptyline or topiramate, as compared to placebo (23).

Verapamil, levetiracetam and zonisamide have also been studied for treatment of migraine, but there is a lack of evidence supporting their use in the pediatric population (34).

### Serotonin Modulators

Pizotifen was studied in a placebo controlled trial conducted on 37 subjects (6–15 years), at a dosage of 1.5 mg/day, with a significant reduction in attack frequency and mild side effects (35). In a subsequent controlled study, the dose of 1–1.5 mg, administered for 6 months in 47 migraine subjects (7–14 years), was not more effective than placebo. Side effects consisted of sedation, increase in appetite and weight (36). In the last decade, no trials have been conducted on pizotifen from which definitive efficacy data can be drawn.

Cyproheptadine was first evaluated in an open study at a dosage of 0.2–0.4 mg/kg/day for 3–6 months, achieving a good improvement (68%) and a remission (21%) of the headache (37). This substance, usually used in younger patients, can have the same side effects as pizotifen, that is drowsiness, weight gain and tenderness. Contraindications consist of asthma, glaucoma and peptic ulcer.

Despite the lack of definitive data, Pizotifen is the only licensed drug in Italy for prophylaxis in migraineurs children (7, 38).

A recent survey on treatments for primary headaches, in 13 specialized juvenile Italian headache centers, reported that pizotifen (1 mg/kg/day) was one of the most efficacious (82% perceived by patients) and tolerated treatments for migraineurs children (12).

## Non-pharmacological Approach

### Nutraceutics and Herbals

The term Nutraceutical refers to all those compounds that derive from “nutrition” and “pharmaceutical.” It refers to the study of active ingredients of food origin that are supposed to have a beneficial function on human health. More active ingredients can be combined with each other to enhance their effects. The term “herbal” refers to all those compounds, such as plants or derivatives of medicinal plants. In general, nutraceutics are chosen to have fewer side effects and a more “natural” approach to the treatment of the disease. These products are generally marked in the absence of validative studies (efficacy and safety) (39).

Data on the use of nutraceuticals and herbals are available for the following molecules: magnesium, riboflavin, coenzyme Q10, butterbur, feverfew and hydroxytryptophan (40).

The rationale of the use of nutraceutics in the treatment of migraine is based on the involvement of these substances in anti-inflammatory or antioxidant molecular pathways or in the mitochondrial energy activity (39).

Despite the widespread use in clinical practice, there are few RCTs available for these substances. Thus, the level of evidence remains low (level b or c), as well as the recommendation (class III).

The few RCTs on magnesium, riboflavin, feverfew, and hydroxytryptophan are prior to 2010 and have not shown conclusive results (41–43).

A more recent RCT investigated the effect of coenzyme Q10 (100 mg/day) in the prophylaxis of pediatric migraine (44). A significant reduction in migraine frequency (*p* < 0.001), severity (*p* < 0.05), and duration (*p* < 0.05) was equally found in the placebo and CoQ10 groups (44).

Ginkolide B, in combination with other nutraceutics, was studied in pediatric open label studies. It is a platelet-activating factor (PAF) receptor antagonist, and would modulate pro-inflammatory mechanisms (42). One open-label trial verified the efficacy of a complex of ginkgolide B, coenzyme Q10, riboflavin and magnesium (doses not specified) in pediatric patients with migraine. After 3 months of treatment, the number of attacks in a month was significantly lower (45). Another open label study compared the efficacy of a combination of ginkgolide B (80 mg/day), coenzyme Q10 (20 mg/day), riboflavin (1.6 mg/day), and magnesium (300 mg/day) with a complex of L-tryptophan (250 mg/day), 5-hydroxytryptophan (50 mg/day), vitamin PP (9 mg/day), and vitamin B6 (1 mg/day) for a treatment period of 6 months. Both combinations were associated with a significant reduction of frequency of headache attacks with a major effect for the complex including ginkgolide B (39, 40).

### Onabotulinumtoxin A

The use of botulinum toxin proved promising in adult patients with migraine, and in particular, its efficacy has been recognized in adults with chronic migraine. However, there are few retrospective data regarding the pediatric experience. This treatment is particularly useful in patients that present side effects of oral drugs or in drug resistant migraine (46). In a retrospective case series study, Ahmed et al evaluated tolerability and efficacy of botulinum toxin type A in the treatment of pediatric chronic headache (47). The study included 10 patients with age ranging from 11 to 17 years who received a standard 100-unit dose of onabotulinumtoxin A. The patients had attempted an average of 8.0 ± 2.40 SD therapies prior to botulinum toxin. A decrease in headache intensity was observed in 40% of patients and 20% noted a decrease in headache frequency with global improvement in quality of life (47). In 2012, Kabbouche et al. reviewed the data of pediatric patients who had received Onabotulinumtoxin A (average dose of 188.5 units±32 with a minimum dose of 75 units and maximum of 200) for chronic migraine in a pediatric headache center from 2004 to 2010. A significant reduction in the frequency of the headache attacks was observed (from 27.4 headache per month±5.2 to 21.3 ± 10.3; *p* < 0.05), while there was no significant change in the severity of pain (48).

### Complementary Therapies

Non-pharmacological treatment for pediatric migraine includes cognitive behavioral therapy, acupuncture, and biofeedback.

As stated above, cognitive behavioral therapy (CBT) proved effective in treating chronic forms of migraine, although the best results were observed when this therapy was combined with pharmacological therapy, in particular amitriptyline (25–28).

A randomized study conducted on 135 patients (mean age 14.4 ± 2) with chronic migraine evaluated the efficacy at 20 weeks of the combined treatment with CBT plus amitriptyline vs. headache education plus amitriptyline. The authors found that 47% of patients in the CBT plus amitriptyline group had less than four headache days per month compared to 20% in the headache education plus amitriptyline group (*p* < 0.005). At 12 months post treatment, 72% of patients in the CBT plus amitriptyline group had less than four headache days per month compared to 52% in the headache education plus amitriptyline group (*p* < 0.05) (27).

In a recent RCT, two different training programs [multimodal cognitive-behavioral training (CBT) and applied relaxation (AR)] were compared with an educational intervention (EDU). Sixty-five children and adolescents with at least 2 attacks of headache per month were assigned to one of the three group. The main outcome endpoints included changes in headache frequency, intensity and duration, responder rate (50% reduction of headache frequency), and number of the attacks needed to treat (NNT). All three groups presented a significant reduction in headache frequency and duration, while no significant differences were observed in the intensity of pain. The group of CBT showed the highest responder rates (50% reduction of headache frequency) after 4 weeks of treatment (63 vs. 32% of AR and 19% of EDU). However, at follow-up after six months, no significant differences were found in the NNTs (CBT: 63%, AR: 56%, EDU: 55%). At follow-up assessment, the effects of the headache frequency remained stable in all groups (49).

There is only limited data on the use of acupuncture for the treatment of pediatric migraine. While efficacy of acupuncture in reducing the frequency of the attacks of migraine was shown in earlier studies (50, 51), no further result has been published in the last 10 years.

Although there are no studies in the last decade on the efficacy of biofeedback for the treatment of pediatric migraine, a recent meta-analysis resumes the main findings on this topic (52). It concludes that biofeedback showed efficacy in reducing attack frequency (*p* < 0.001) and duration (*p* < 0.001), and intensity of pain (*p* < 0.001). However, biofeedback demonstrated no adjuvant effect when combined with other behavioral and no more benefits than pharmacological treatment (52). It is worth to be underlined that data on biofeedback comes only from retrospective studies or pilot studies (53–55).

Overall, non-pharmacological treatment for migraine can be a valid alternative for selected patients.

The choice of a non-pharmacological therapy should be reserved for patients who have failed drug therapies or, as a first line treatment, in patients who cannot tolerate the side effects of drugs. However, most published studies on non-pharmacological treatments have been carried out in adults, while definite results in children and adolescents are still lacking. Therefore, further confirmation with rigorous randomized controlled trials is mandatory for the majority of these approaches (56).

## Further Considerations and Future Prospectives

The main novelty of the last decade in the prophylaxis of pediatric migraine comes from the results of the CHAMP study. This study showed that pharmacological treatments, such as amitriptyline and topiramate, do not differ from placebo. Three main issues are raised by this study:

-*First, placebo effect proves very powerful in pediatric age (about 60% of patients), thus it should be considered as a fundamental therapeutic resource*. Placebo response rate is known to be high in pediatric migraine studies (25). The high therapeutic efficacy of placebo should not be considered only as a threat to the success of clinical studies, but it represents a therapeutic possibility in the treatment of pediatric migraine. Research should be addressed to further investigate the exact mechanisms connected with high placebo response rate in children with migraine. A higher knowledge in this field could allow us to use placebo as a non-harmful and effective treatment.
- *The CHAMP study get us to wonder whether the use of pharmacological treatment is still allowed*. Although the CHAMP results must be taken into account, we cannot forget the results of other RCTs reviewed in the present study and supporting the efficacy of some pharmacological treatments. Moreover, CHAMP trial did not consider some dynamics that may influence the course of migraine independently of drug therapy, such as psychological factors mostly linked to school attendance. It is known that untreated young migraineurs have a lower frequency of attacks in summer months, while they suffer more after the start of the school (57, 58). This means that whether the efficacy of placebo is measured in a favorable (e.g., from February to August), or unfavorable (e.g., from August to February) period can influence the response to therapy. In conclusion, we believe that CHAMP study should induce us to be even more rigorous in the treatment selection, considering the evidence-based data of efficacy and safety as being crucial for the therapeutic choice.
- *Lastly, we must underline that there is no drug available in pediatric age with exclusive indication for migraine treatment* (59). From this point of view, there is high expectation for the use of calcitonin gene-related peptide (CGRP) inhibitors (CGRP-r). The large trials conducted in the adult population (60, 61) have led Food and Drug Administration (FDA) to give the green light to commercialization of these drugs (Erenumab; Galcanezumab; Fremanezumab) in USA. The same drugs have been recently approved from European Medicines Agency (Erenumab; Galcanezumab). Although results from trials in children and adolescents are not available yet, the Pediatric and Adolescent Headache special interest group of the American Headache Society proposed recommendations on the use of these agents for pediatric headache disorders (62). The authors suggested that the use of CGRP receptor antagonists could be considered in postpubertal adolescent patients with frequent migraine attacks (≥8 headache days/month), who have moderate to severe disability associated with migraine (PedMIDAS score ≥30) and have failed ≥2 preventive therapies. For younger patients, who are refractory to multiple preventive therapies, CGRP receptor antagonists may also be considered with proper monitoring (e.g., bone health, linear growth, weight/BMI, infections) (62).

## Author Contributions

LP and FU took care of the selection of the articles and wrote the manuscript. RM and ST contributed to the selection of articles. MF and GS contributed to the methodology. FV supervised the final manuscript. MV designed and supervised the work.

### Conflict of Interest Statement

The authors declare that the research was conducted in the absence of any commercial or financial relationships that could be construed as a potential conflict of interest.
